# Increasing Detection of Legionnaires’ Disease in a Large Italian Hospital in the Period 2016–2023

**DOI:** 10.1007/s44197-024-00276-8

**Published:** 2024-07-18

**Authors:** Marilena La Sorda, Flavio De Maio, Maria Scaturro, Barbara Fiori, Giulia Santarelli, Jessica Iera, Fabiola Mancini, Brunella Posteraro, Maria Luisa Ricci, Maurizio Sanguinetti

**Affiliations:** 1 Department of Laboratory and Infectious Sciences, Fondazione Policlinico Universitario A. Gemelli IRCCS, L.go A. Gemelli 8, 00168 Rome, Italy; 2 Department of Infectious Diseases, Istituto Superiore di Sanità, Rome, Italy; 3 Department of Basic Biotechnological Sciences, Intensive and Perioperative Clinics, Università Cattolica del Sacro Cuore, L.go F. Vito 1, 00168 Rome, Italy; 4 Department of Abdominal and Endocrine Metabolic Medical and Surgical Sciences, Fondazione Policlinico Universitario A. Gemelli IRCCS, L.go A. Gemelli 8, 00168 Rome, Italy; 5 ESCMID Study Group for Legionella Infections (ESGLI),, Basel, Switzerland

**Keywords:** Legionnaires’ disease, *Legionella pneumophila*, Diagnostic methods

## Abstract

The pandemic marked the beginning of an era of dynamic and rapid changes in the diagnosis of respiratory infections. Herein we describe Legionnaires’ disease trend in the years 2016–2023 in a large Italian hospital showing how improvements in diagnostic algorithms impact on its detection.

## Correspondence

A range of different clinical manifestations caused by *Legionella* are defined as Legionellosis, from a flu like illness named Pontiac fever to Legionnaires’ disease (LD). LD, caused mainly by *Legionella pneumophila*, is a severe and often fatal pneumonia classified as community acquired, travel associated, or hospital acquired according to the type of exposure [1]. Indeed, *Legionella* is usually found at low concentrations in natural freshwater environments and, in favorable conditions, it is capable of multiplying and passing into man-made water systems and to be transmitted via inhalation of contaminated water droplets [2].

The recent pandemic turned the spotlight on the validity and appropriateness of numerous laboratory procedures focusing primarily on the diagnosis of respiratory infections including LD. On the other side, COVID-19 pandemic led to renewed interest in respiratory infections and yielded improvements in diagnostic processes that sometimes highlighted important changes in disease seasonality or differences in susceptibility for some subjects, such as that found for respiratory viruses or *S. pyogenes* [4–5].

To date, the diagnosis of LD has mainly relied on urinary antigen detection, while culture of lower respiratory tract secretions, serology and PCR are seldom adopted [2, 3]. Ideally, the diagnosis of LD is conducted using as many methods as possible because each has some limitations. Urinary antigen detection, for example, predominantly detects serogroup 1; serological tests require the collection of paired acute and convalescent samples; while for culture it is difficult to obtain an adequate sample and early antibiotic therapy may affect the analytical result; methods based on *Legionella* DNA detection are not yet routinely adopted because they do not define a confirmed LD case. [7–8].

The aim of this study is to analyse data on LD diagnosis over an 8-year period (2016–2023) at one of the largest hospitals in Italy (Policlinico Agostino Gemelli, Rome, Italy) obtained through various diagnostic assays.

**Fig. 1:**
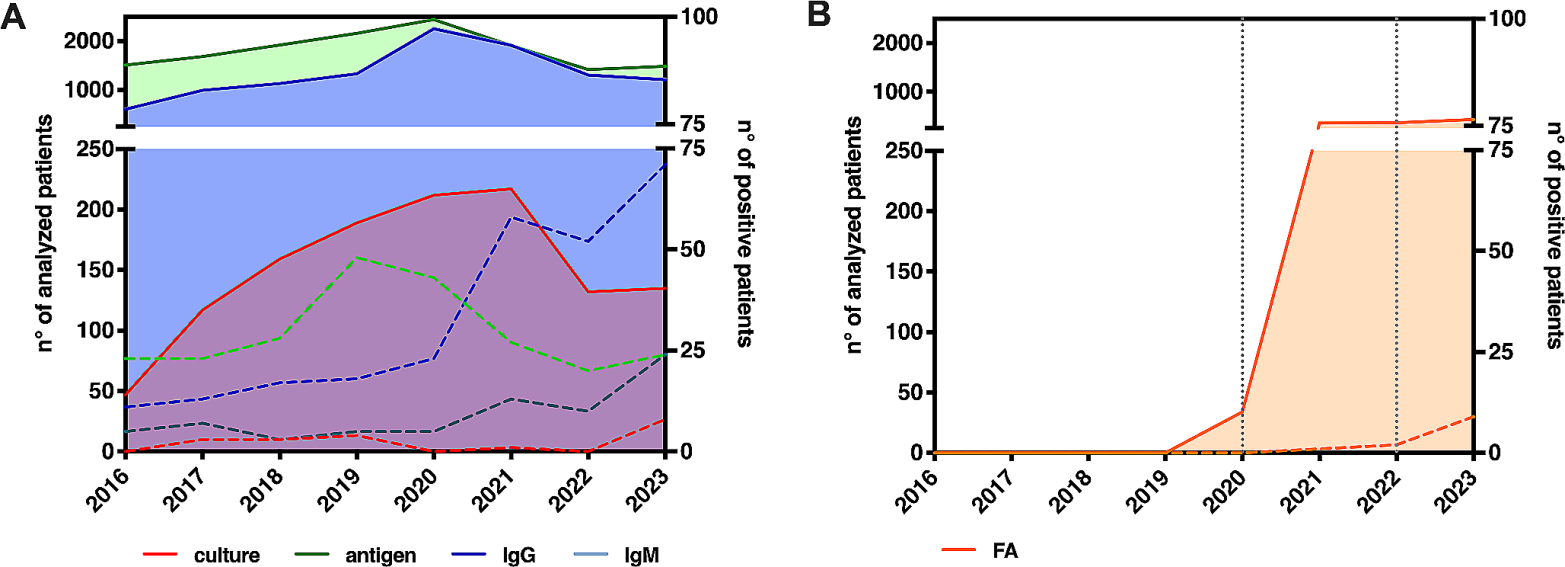
Schematic representation of the number of specimens analyzed for *Legionella pneumophila*. **A**. Continuous lines and area under the lines show number of samples analyzed for *Legionella pneumophila* using culture method (red), urinary antigen (green) and immunoglobulins (IgG and IgM, blue and cyan, respectively) (left Y axis). Dotted lines describe positive samples (right Y axis). **B**. Representation of molecular test carried out for detection of *Legionella pneumophila* (continuous orange line and area under the line; left Y axis). Orange dotted line shows positive specimens (right Y axis), whereas gray dotted lines refer to the starting use of FA on respiratory samples (2020) and to laboratory care availability 24 h a day, 7 days a week (2023)

A total of 1208 respiratory samples (consisting of BAL, sputum or pharyngeal swabs) were obtained and analyzed by culture method, using both selective (BMPA, MWY, GVPC, Oxoid, Thermofisher) and unselective (BCYE, Oxoid, Thermofisher) agar media; while 14,528 urine specimens were analyzed for *Legionella* antigen detection (STANDARDTM F Legionella Ag FIA, SD Biosensor), able to detect serogroups 1, 3, 5, 6, 8 (Table 1; Fig. 1A). On the other side, anti-Legionella Immunoglobulin G and M were probed for 10,747 and 10,761 samples, respectively, by using Anti-Legionella pneumophila (IgG-Ig M) ELISA (Euroimmun, Germany) (Table 1; Fig. 1A). Clinical isolates were finally typed by monoclonal subgroup using the Dresden monoclonal antibodies panel and by sequence-based typing to determine the sequence type (ST) [7, 8]. Biofire Filmarray Pneumonia plus (FA, Biomerieux) was used for molecular detection of *Legionella pneumophila* (Lp).

**Table 1:** Samples analyzed for *Legionella pneumophila* detection in the period 2016–2023. Number of analyzed samples and results obtained using cultural method, antigen detection, molecular assay and serology

		Culture		Urinary antigen		Molecular assay		Antibodies (IgG)		Antibodies (IgM)	
		n°	%	n°	%	n°	%	n°	%	n°	%
**2016**	Inconclusive	-	-	-	-			7	1,2	1	0,2
	negative	47	100,0	1485	98,5			586	97,0	603	99,0
	positive	-	-	23	1,5			11	1,8	5	0,8
	Total	47		1508				604		609	
**2017**	Inconclusive	-	-	-	-			27	2,7	1	0,1
	negative	114	97,4	1662	98,6			952	96,0	977	99,2
	positive	3	2,6	23	1,4			13	1,3	7	0,7
	Total	117		1685				992		985	
**2018**	Inconclusive	-	-	-	-			31	2,7	32	2,8
	negative	157	98,7	1893	98,5			1086	95,8	1092	96,9
	positive	2	1,3	28	1,5			17	1,5	3	0,3
	Total	159		1921				1134		1127	
**2019**	Inconclusive	-	-	-	-			43	3,2	5	0,4
	negative	186	98,4	2110	97,8			1268	95,4	1320	99,2
	positive	3	1,6	48	2,2			18	1,4	5	0,4
	Total	189		2158				1329		1330	
**2020**	Inconclusive	-	-	-	-	-	-	43	1,9	5	0,2
	negative	212		2399	98,2	34	100,0	2187	97,1	2260	99,6
	positive	-	-	43	1,8	0	0,0	23	1,0	5	0,2
	Total	212		2442		34		2253		2270	
**2021**	inconclusive	-	-	-	-	-	-	78	4,1	12	0,6
	negative	216	99,5	1882	98,6	360	99,7	1781	92,9	1894	98,7
	positive	1	0,5	27	1,4	1	0,3	58	3,0	13	0,7
	Total	217		1909		361		1917		1919	
**2022**	inconclusive	-	-	-	-			42	3,2	29	2,2
	negative	132	100,0	1401	98,6	363	99,5	1214	92,8	1265	97,0
	positive	-	-	20	1,4	2	0,5	52	4,0	10	0,8
	Total	132		1421		365		1308		1304	
**2023**	inconclusive	-	-	-	-			68	5,6	30	2,5
	negative	131	97,0	1460	98,4	422	97,9	1071	88,5	1163	95,6
	positive	4	3,0	24	1,6	9	2,1	71	5,9	24	2,0
	Total	135		1484		431		1210		1217	

In the years 2019 and 2021 we noted the maximum peak of cultural exams, which was not associated with an increase of *Legionella* positive samples. In 2017, 2018 and 2019 the incidence of positive *Legionella* spp. culture was stable with cultures being positive for 2.6%, 2.1% and 2.1% of samples respectively. During the pandemic, incidence of culture proven LD plummeted reaching values as low as 0 positives despite an increase in the number of tested specimens. Interestingly, in 2023 this value climbed to 5.9%, although the number of performed tests was comparable to pre-pandemic years. Conversely, there were no differences in the positivity rates of urinary antigens across the analyzed period. This discrepancy may be attributed to the preventive administration of azithromycin in patients with pneumonia regardless of the confirmed diagnosis of COVID-19 which may have inhibited the permanence of *Legionella* in respiratory secretions but allowed the release of urinary antigen [9, 10]. Similarly, the number of requested serological tests for LD slightly increased in the years 2020–2021, even though no meaningful increase in positive specimens was detected.

An increased rate of culture positive samples was detected in 2023 (8/135), whereas antigen detection was stable in the studied period, except for the period 2019–2020 which had twice as many positive samples as previous years, this was related to an increased number of tests and there was no significant change in the positivity rate.

The combination of molecular and culture assays leads to the detection of 26 LD cases: 7 FA positive, 11 culture positive and 8 positive specimens for both methods. Of note, 6 of the 7 FA positive samples were pharyngeal swabs. Culture allowed for the isolation of *Legionella pneumophila* from respiratory samples of 15 patients (68% community, 18% nosocomial, 14% travel associated). The strains were all serogroup 1 (Lp1) except for 5 isolates (Lp5, Lp6 and three Lp10). Lp1 belongs mainly to the more virulent class of monoclonal subgroup (seven of ten were MAb3/1 positive) [9].

STs were determined for all the isolates except one Lp10 isolate, for which it was not possible to determine the *neuA* allelic number. Other strains showed the following STs: 1 (two isolates), 20, 42, 80, 143, 476, 700, 758 (two isolates), 824, 1260, 1333 and 1362.

The culture method is still considered the gold standard for LD diagnosis, despite several technical limitations in the identification of non *Legionella pneumophila* species prompt to consider molecular approaches. In the last years the introduction of DNA based detection assays that, although expensive, have enhanced in particular the diagnosis of difficult to cultivate bacteria. Our data highlight an improvement in the detection of *Legionella* thanks to the introduction of FA, from 2020 to 2023, allowing to detect 12 additional positive samples with a peak (*n* = 9) in 2023.

In this scenario, we record two significant changes that may have impacted on LD diagnosis: (a) the extensive use of FA, upon specific request by clinicians; (b) the laboratory care available 24 h a day, 7 days a week (Fig. 1B). These factors, combined with early biomarkers (CRP, LDH, PCT, PLTs count, hyponatremia, fever and cough without sputum) bring to improve LD diagnosis with the isolation and characterization of the pathogen. Of note, FA was achieved only when a severe pneumoniae was clinically suspected or radiologically diagnosed allowing to rapidly identify causative etiological agents [11]. Importantly, Lp DNA positive samples, as well as culture positive samples were all associated to a severe pulmonary disease depicting LD. Unfortunately, *Legionella* isolation remains the only method to characterize at genomic level the bacterium in order to identify the possible source of infection and implement the microbiological and epidemiological surveillance (spread of particular STs, outbreaks, antimicrobial susceptibility, etc.).

In this context, metagenomics may be a future promising tool to genetically characterize isolates, due to its high sensitivity and working directly on clinical specimens.

More clarity is needed on the serological data so that IgM and IgG might potentially aid in the management of Legionella-related pneumonia. However, frequently, paired tests are unavailable or unreliable due to a cross-reactivity.

**Fig. 2:**
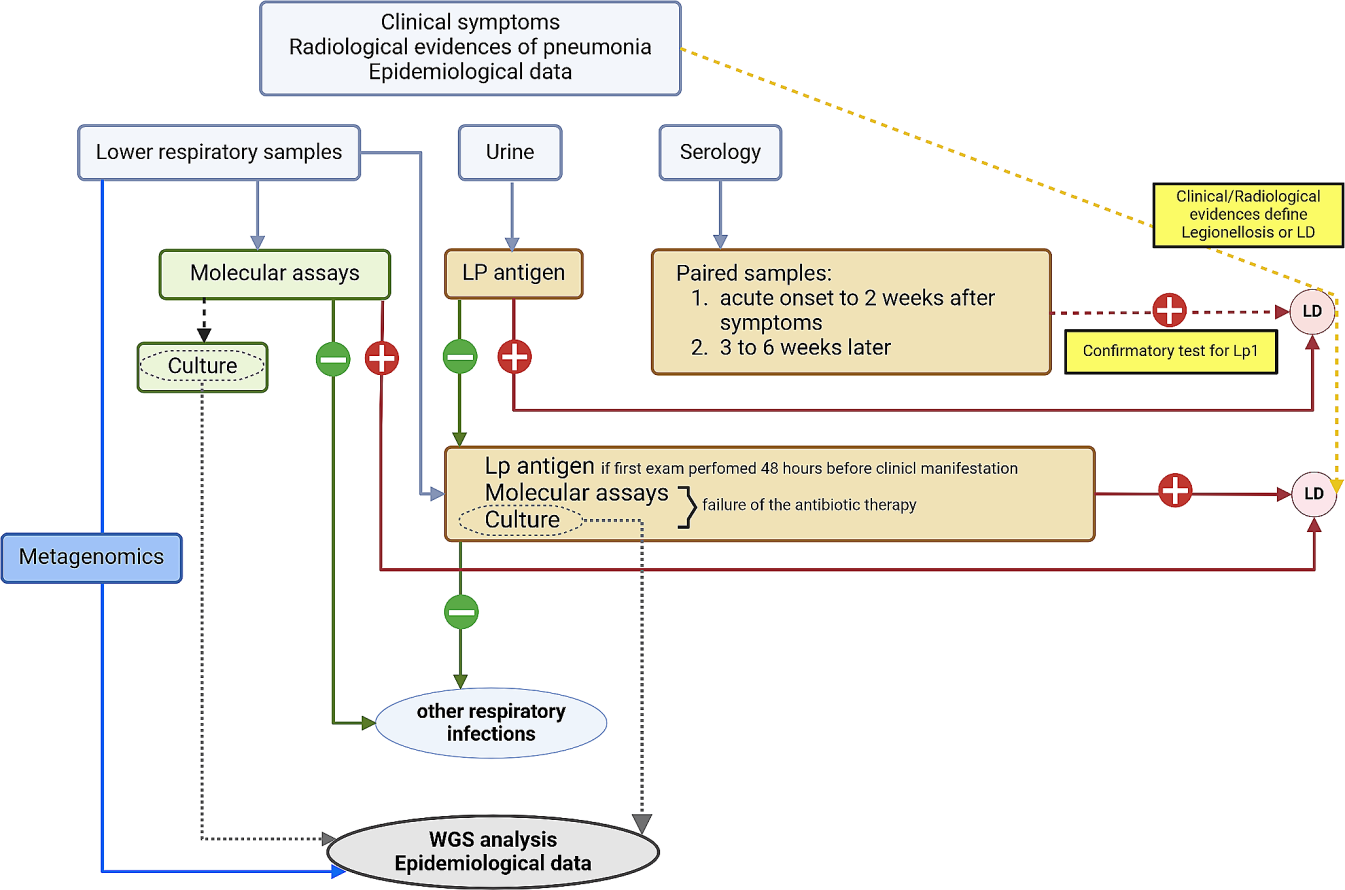
Schematic representation of the processes to detect *Legionella pneumophila* infection by searching for urinary antigen, culture or molecular tests (orange boxes as presented by Cattan and colleagues [7]). Green boxes highlight the improved diagnostic flowchart starting from lower respiratory specimens and applying molecular assay which affects culture method. Whole genome sequencing (WGS) for the ultimate epidemiological analysis is based on the cultural isolation of the pathogen. Metagenomics (blue boxes) may represent a novel and promising tool to acquire more information regarding *Legionella pneumophila*,and other *Legionella* spp., genomics and epidemiology

We did not investigate specific clinical data, but our study was mostly focused on the complementary use of different diagnostic tests and to a continuous working flowchart as resumed in Fig. 2. Indeed, our data do not highlight a significant increase rate of LD disease in the post COVID-19 pandemic, conversely the major isolation of the pathogen may be related the adoption of *Legionella* DNA detection used as a wake-up call method (i.e. plating the respiratory secretions in many plates) increasing the probability of *Legionella* detection.

## Data Availability

No datasets were generated or analysed during the current study.
